# Genome-Informed Identification of Species-Specific Diagnostic Markers for Listeria Using Pangenome Analysis

**DOI:** 10.3390/pathogens15040397

**Published:** 2026-04-07

**Authors:** Viona Osei, Emmanuel Kuufire, Rejoice Nyarku, Kingsley E. Bentum, Tyric James, Asmaa Elrefaey, Temesgen Samuel, Woubit Abebe

**Affiliations:** 1 Center for Food Animal Health, Food Safety and Defense, Department of Pathobiology, College of Veterinary Medicine, Tuskegee University, Tuskegee, AL 36088, USA; vosei3882@tuskegee.edu (V.O.); ekuufire9436@tuskegee.edu (E.K.); rnyarku8794@tuskegee.edu (R.N.); kbentum8786@tuskegee.edu (K.E.B.); tjames2981@tuskegee.edu (T.J.); aelrefaey3754@tuskegee.edu (A.E.); tsamuel@tuskegee.edu (T.S.); 2 Department of Food Hygiene and Control, Faculty of Veterinary Medicine, University of Sadat City, Sadat City 32897, Egypt

**Keywords:** *Listeria*, pangenome analysis, whole-genome sequencing, molecular diagnostics, foodborne pathogens, species-specific targets

## Abstract

The genus *Listeria* comprises diverse bacteria with significant public health relevance, particularly *Listeria monocytogenes*. A comparative genomic analysis of ten representative *Listeria* species was conducted using 33 high-quality genome assemblies to investigate core and accessory genome dynamics and identify candidate diagnostic loci. Pangenome reconstruction was performed using the Roary Integer Linear Programming Bacterial Annotation Pipeline (RIBAP) to classify core, soft-core, and accessory genes, while average nucleotide identity (ANI) analysis assessed genomic relatedness across thresholds of 60–95%. Functional annotation of core and species-specific genes was conducted using Genome Annotation and Information Analysis (GAIA). Core genes were highly conserved and associated with essential cellular functions, whereas the accessory genome contributed to species-level diversification and ecological adaptation. Candidate molecular markers were derived from accessory genes and evaluated based on presence/absence across genomes, retaining loci present in ≥80% of target strains and absent in non-target strains. Experimental validation of selected primers was performed using two *L. monocytogenes* reference strains (ATCC 19117 and ATCC BAA-679) with conventional PCR and gel electrophoresis to confirm expected amplicon sizes and specificity. These findings establish a genome-informed, specificity-driven framework for marker development and highlight the accessory genome as a valuable source of diagnostic loci, supporting accurate detection, epidemiological surveillance, and food safety monitoring.

## 1. Introduction

The genus *Listeria* comprises a diverse group of Gram-positive bacteria widely distributed across natural and anthropogenic environments, including soil, water, vegetation, food-processing facilities, and animal reservoirs [1,2]. Taxonomically, *Listeria* belongs to the phylum Firmicutes, class Bacilli, order Bacillales, and family Listeriaceae [3]. To date, approximately 28 species have been described within the genus, including *L. aquatica*, *L. booriae*, *L. cornellensis*, *L. costaricensis*, *L. cossartiae*, *L. farberi*, *L. fleischmannii*, *L. floridensis*, *L. goaensis*, *L. grandensis*, *L. grayi*, *L. ilorinensis*, *L. immobilis*, *L. innocua*, *L. ivanovii*, *L. marthii*, *L. monocytogenes*, *L. newyorkensis*, *L. portnoyi*, *L. riparia*, *L. rocourtiae*, *L. rustica*, *L. seeligeri*, *L. swaminathanii*, *L. thailandensis*, *L. valentina*, *L. weihenstephanensis*, and *L. welshimeri* [4,5]. Among these, *L. monocytogenes* and *L. ivanovii* are currently the only species known to be pathogenic to humans and animals, sharing numerous virulence factors and mechanisms of pathogenicity [3,4]. Of the two species, *L. monocytogenes* exhibits greater pathogenic potential, causing systemic infections, meningitis, and pregnancy-associated listeriosis in humans, as well as encephalitis, septicemia, and abortions in ruminants [6,7]. In contrast, *L. ivanovii* primarily affects ruminants, leading to stillbirths and fetal loss, while human infections remain rare [8]. Conversely, *L. innocua* is reported as the most frequently isolated species within the genus *Listeria* [9].

*L. monocytogenes* is of particular public health concern due to its ability to cause listeriosis, a severe foodborne disease associated with high hospitalization and mortality rates, especially among immunocompromised individuals, pregnant women, neonates, and the elderly [10,11]. In 2025, the United States Centers for Disease Control and Prevention (CDC) estimated that approximately 1250 cases of listeriosis occur annually in the United States, resulting in nearly 172 deaths per year and underscoring the substantial public health burden posed by this pathogen [12]. Despite its relatively low incidence compared to other foodborne pathogens [3], *L. monocytogenes* is recognized as one of the four priority foodborne pathogens of global public health concern [13].

Consequently, the development and implementation of accurate and sensitive detection methods across the entire food production chain, from farm to fork, are essential for effective pathogen surveillance and for enabling early detection and control of foodborne illnesses [14]. Conventional detection approaches, including culture-based and biochemical methods, remain widely used due to their simplicity and cost-effectiveness [15,16]. However, these methods are labor- and time-intensive and may be compromised by inhibitory food matrices, limited specificity, and false negative results [4,14,17]. To address these limitations, molecular diagnostic approaches have been increasingly adopted as complementary tools, exploiting genomic variation rather than solely phenotypic expression to enhance detection accuracy and reliability, and are typically used alongside conventional microbiological methods [14,16].

Polymerase chain reaction (PCR)-based assays, which target specific genetic regions to detect, differentiate, and quantify pathogens, have become indispensable tools in food safety surveillance and clinical diagnostics [15,16,17]. By targeting highly specific and evolutionarily conserved genomic regions, PCR enhances sensitivity, specificity, and reproducibility and has been widely applied for the differentiation of *Listeria* species and *L. monocytogenes* serotypes [14,15,16].

Advances in whole-genome sequencing (WGS) have further transformed the study of foodborne pathogens, including *Listeria*, by enabling high-resolution analyses of population structure, virulence, ecological adaptation, and evolutionary dynamics [9,16]. WGS is increasingly employed to investigate outbreak dynamics, persistence within food-processing environments, and phylogenetic relationships among *Listeria* species [15,16]. Moreover, WGS facilitates in silico prediction of antimicrobial resistance determinants, virulence factors, and stress-adaptation traits that contribute to *Listeria* survival along the food supply chain [15]. Integration of WGS with advanced bioinformatic frameworks has enhanced the precision, reproducibility, and scalability of comparative genomic analyses, positioning WGS as a cornerstone technology for high-resolution surveillance and proactive management of *Listeria*-associated foodborne risks [15,18].

Beyond these applications, pangenome-informed analyses provide a powerful framework for exploring genomic diversity by partitioning the total gene repertoire of a bacterial genus into core, accessory, and strain-specific components [15,19]. The core genome, conserved across all strains, underpins essential cellular functions, whereas the accessory genome, including shell and cloud genes, drives intraspecies diversity and confers selective advantages such as antimicrobial resistance, virulence, and environmental adaptation [16,18,19]. In *Listeria*, pangenome studies have revealed extensive genetic variability and underscored the role of accessory genes in shaping ecological fitness, species differentiation, and pathogenic potential [15,18,20]. Recent studies have expanded the taxonomic and genomic landscape of the *Listeria* genus through refined species characterization and the growing availability of high-quality genome sequences [4,9]. Importantly, the identification of species-specific genetic markers derived from pangenome analyses enables the development of sensitive and specific molecular assays for accurate detection and characterization of foodborne pathogens in complex matrices, thereby strengthening monitoring strategies and reducing contamination risks in food production systems [15]. Advances in functional annotation platforms have further facilitated exploration of conserved and species-specific genes, providing deeper insight into the biological roles of core and accessory genomes [21].

The objectives of this study were to perform a comprehensive pangenome analysis of ten *Listeria* species using high-quality genome assemblies and to evaluate genomic diversity across the genus by integrating pangenome reconstruction using the Roary Integer Linear Programming Bacterial Annotation Pipeline (RIBAP), average nucleotide identity (ANI) analysis, and functional annotation. The study also aimed to characterize the composition and distribution of core, soft-core, and accessory gene families, and to identify candidate species- and genus-specific loci with potential applications in molecular diagnostics and food safety surveillance.

## 2. Materials and Methods

### 2.1. Genome Acquisition and Curation

FASTA-formatted complete genomic sequences of 35 *Listeria* strains representing 15 species [4] were downloaded from the National Center for Biotechnology Information (NCBI) Nucleotide database on 23 December 2024. Each species included between one and five strains. Genome quality was evaluated using CheckM (v1.0.18) [22], applying thresholds of ≥90% completeness, ≤5% heterogeneity, and ≤5% contamination. After quality control, 33 genomes representing 10 species were selected for subsequent analyses.

The genomes included: *L. aquatica* (1), *L. grayi* (3), *L. innocua* (5), *L. ivanovii* (5), *L. marthii* (2), *L. monocytogenes* (5), *L. newyorkensis* (1), *L. seeligeri* (5), *L. weihenstephanensis* (1), *L. welshimeri* (5); species names and accession numbers are provided in Table 1.

### 2.2. Data Analysis

#### 2.2.1. Pangenome Reconstruction and Core/Accessory Gene Analysis

The curated genomes were subjected to pangenome reconstruction using the RIBAP [19] integrated within a Nextflow workflow framework to ensure computational reproducibility [23]. Initial gene annotation was performed with Prokka, and orthologous gene clusters and core genome alignment were generated using Roary [24]. RIBAP applies an integer linear programming algorithm to optimize gene clustering beyond standard Roary predictions, enabling precise classification of core, soft-core, and accessory gene sets [19].

Genomic relatedness was further evaluated across a range of ANI thresholds (60%, 70%, 80%, 90%, 95%). The selected ANI thresholds are consistent with widely accepted genomic delineation criteria for bacterial taxa, where ≥95% ANI approximates species boundaries [25,26].

**Figure 1:**
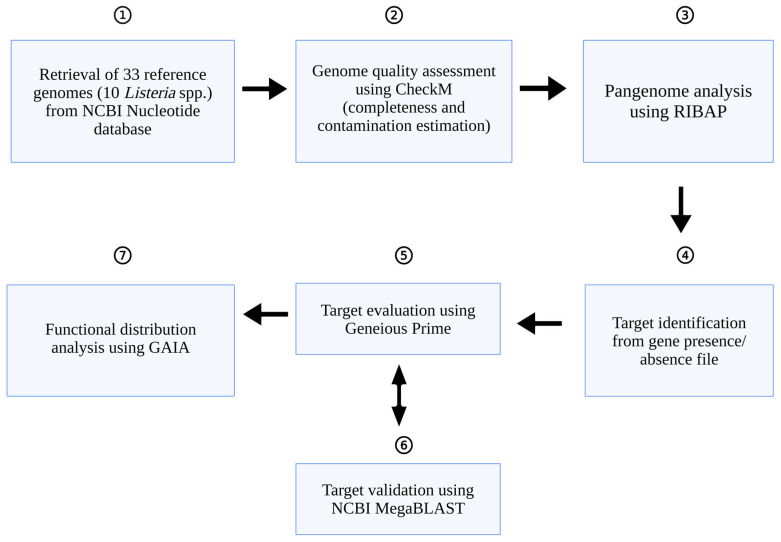
Schematic overview of the analytical workflow. This figure was created with Available online: BioRender.com (accessed on 25 February 2026).

High-resolution species delineation was achieved at 95% ANI, whereas lower thresholds facilitated broader phylogenetic comparisons. Pangenome composition and ANI-based clustering were visualized using Phandango [27]. A schematic overview of the workflow is provided in Figure 1.

#### 2.2.2. Identification and In Silico Validation of Species-Specific Targets

Accessory genes unique to each *Listeria* species were extracted from the RIBAP-generated gene presence/absence matrix. Candidate sequences were examined in Geneious Prime (2024.0.2) to confirm their distribution across strains and sequence integrity. In silico specificity was assessed by comparison against the NCBI non-redundant nucleotide database (nr/nt) using both the built-in Basic Local Alignment Search Tool (BLAST) tool in Geneious Prime and NCBI MegaBLAST. Candidate loci were retained if present in the majority of target genomes (typically ≥80–100%) and absent in all non-target genomes within the curated dataset, as determined by BLASTn analysis (identity ≥ 90%, coverage ≥ 90%, E-value ≤ 1 × 10^−10^).

To illustrate species-level gene distribution patterns, randomly selected species-specific genes derived from the same RIBAP-generated gene presence/absence matrix were matched and visualized using Interactive Tree of Life (iTOL) v5 [28], with midpoint rooting applied for visualization (accessed 10 July 2025).

#### 2.2.3. Functionality of Core and Species-Specific Genes

Core genes (95% ANI) and validated species-specific targets were functionally characterized using the Genome Annotation and Information Analysis (GAIA) platform [21]. GAIA was accessed on 14 July 2025, prior to its replacement by SeqHub. Functional assignments were inferred from sequence homology to curated reference databases to provide insight into conserved and species-specific roles in the bacterial genome.

### 2.3. Primer Development and Validation

Highly conserved core and species-specific gene sequences identified through in silico analyses were used as templates for primer design to enable accurate detection of *Listeria* species. Primer pairs were designed using Geneious Prime, with parameters optimized to ensure appropriate melting temperature (Tm), GC content, amplicon size, and minimal secondary structure formation. Primer sequences and full design specifications are proprietary to QuantiPATH and are therefore not disclosed. To ensure scientific transparency, primer design constraints, amplicon size ranges, and validation criteria are fully described.

Primer specificity was experimentally validated using two *L. monocytogenes* reference strains, American Type Culture Collection (ATCC) 19117 and ATCC BAA-679 (EGD-e). Lyophilized cultures were reconstituted in 5 mL of Buffered *Listeria* Enrichment Broth (BLEB) and incubated at 37 °C for 48 h under aerobic conditions. Genomic DNA was extracted using reagents from QIAGEN in accordance with the manufacturer’s instructions. DNA concentration and purity were assessed using the Thermo Scientific™ NanoDrop™ 2000c Spectrophotometer (Thermo Fisher Scientific, Waltham, Massachusetts, USA) prior to subsequent analyses.

Conventional PCR was performed using an Eppendorf Mastercycler^®^ nexus gradient terminal (Eppendorf SE, Hamburg, Germany) in a final reaction volume of 10 µL containing the designed primers, template DNA, and appropriate PCR master mix components. PCR reactions were performed using a commercially available PCR master mix according to the manufacturer’s instructions. Each primer set was tested individually with both ATCC strains. Thermal cycling conditions consisted of an initial denaturation at 95 °C for 3 min, followed by 30 cycles of denaturation at 95 °C for 30 s and annealing at 64 °C for 30 s, with a final extension at 72 °C for 30 s and a hold at 4 °C.

Amplification products were separated by electrophoresis on 1.8% (*w*/*v*) agarose gel prepared in 1× TAE buffer. DNA bands were visualized using a Gel Doc™ EZ imager (Bio-Rad Laboratories, Inc., Hercules, California, USA) to confirm expected amplicon sizes and evaluate primer specificity.

## 3. Results

### 3.1. Core and Accessory Genome Composition

Pangenome analysis of 33 high-quality *Listeria* genomes revealed pronounced genomic diversity across species. The number of core genes varied considerably across ANI thresholds, ranging from 16 at 95% ANI to 856 at 60% ANI. At the most stringent 95% ANI threshold, 16 highly conserved core genes were identified, including *mnmE*, *ndoA*, *prs_1*, *rplN*, *rplP*, *rpmA*, *rpmB*, *rpmG2*, *rpmJ*, *rpsJ*, *rpsO*, *rpsS*, *sigA*, *spxA*, and *rpoA*, along with one hypothetical protein. These core genes were used for genomic characterization and not for diagnostic assay development.

**Figure 2:**
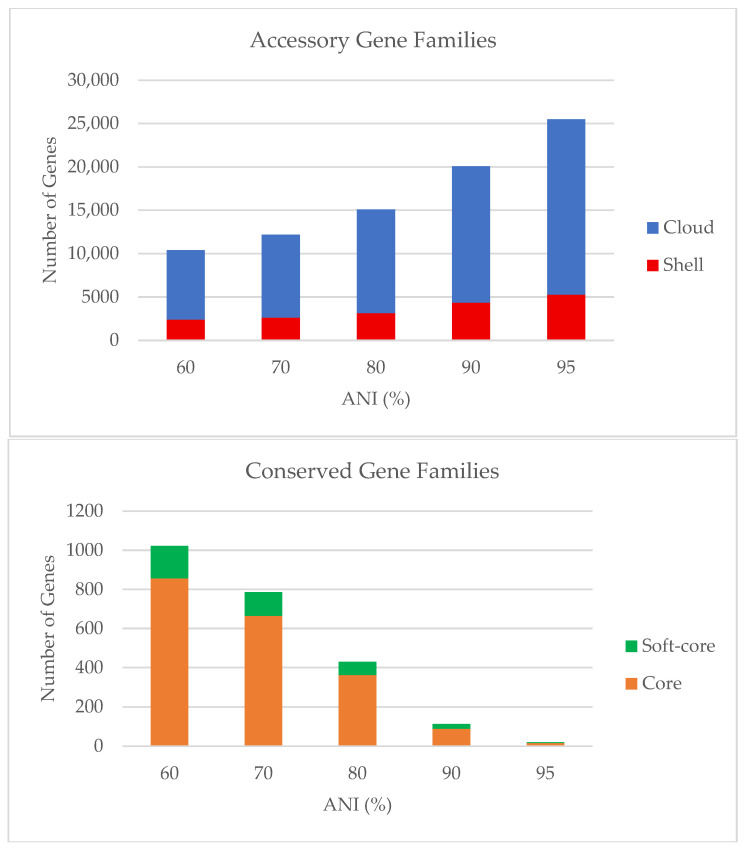
Distribution of conserved and accessory gene families across ANI thresholds in the *Listeria* genus. Gene category counts were derived from Table 2. The (**upper panel**) shows accessory gene families partitioned into shell (red) and cloud (blue) genes as ANI thresholds increase. The (**lower panel**) displays conserved gene families as stacked bars of core (orange) and soft-core (green) genes. Gene counts are plotted separately in two panels to accommodate the large difference in magnitude between accessory and conserved gene categories. ANI thresholds range from 60% to 95% as indicated on the x-axis, with corresponding gene family counts shown on the y-axis.

**Figure 3:**
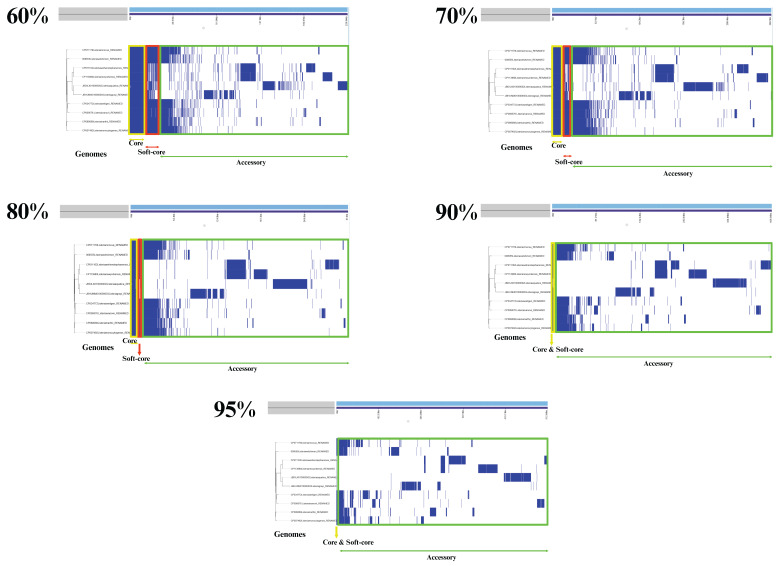
Gene presence–absence matrices across ANI thresholds of 60%, 70%, 80%, 90%, and 95%, visualized using the Phandango platform. Gene presence is indicated in blue and absence in white. Core genes are outlined in yellow, soft-core genes in red, and accessory genes in green. The matrices demonstrate a progressive reduction in shared gene content and increased genomic differentiation among *Listeria* species as ANI thresholds become more stringent.

In contrast, the accessory genome displayed substantial variability among strains, expanding progressively from 10,400 genes at 60% ANI to 25,486 genes at 95% ANI. The detailed distribution of core, soft-core, and accessory gene categories across ANI thresholds is summarized in Table 2 and illustrated in Figure 2 and Figure 3.

### 3.2. Specific Genetic Targets and In Silico Validation (Sensitivity and Specificity)

Accessory gene analysis enabled the identification of putative species-specific targets for each *Listeria* species. Most targets corresponded to hypothetical proteins with currently uncharacterized functions. In silico validation demonstrated high diagnostic potential, with selected targets achieving ≥90% sensitivity and 98–100% specificity across the evaluated strains. Details of the identified targets are summarized in Table 3.

### 3.3. Functional Characterization of Core and Species-Specific Genes

Functional annotation of the conserved core genome using the GAIA platform revealed that core genes are primarily associated with essential cellular processes, including translation, transcriptional regulation, and stress response. At the 95% ANI threshold, the identified core genes were classified under molecular functions, biological processes, and cellular components related to ribosomal structure, protein synthesis, and regulatory activities.

**Figure 4:**
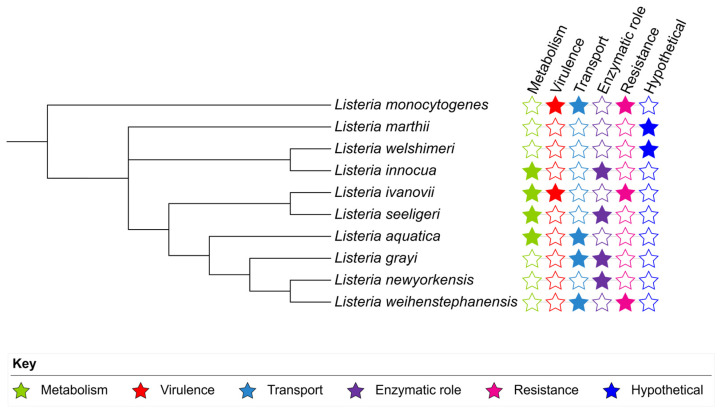
Distribution of randomly selected species-specific genes across ten *Listeria* species visualized on a phylogenetic tree using iTOL v5. Colored stars indicate predicted functional categories of the genes: metabolism (green), virulence (red), transport (light blue), enzymatic role (purple), resistance (pink), and hypothetical proteins (dark blue). Solid stars indicate gene presence, and hollow stars indicate gene absence.

Species-specific targets from the accessory genome were predominantly annotated as hypothetical proteins (Figure 4).

### 3.4. PCR Primer Validation

The specificity and performance of the designed PCR primers were evaluated using two *L. monocytogenes* reference strains, ATCC 19117 and ATCC BAA-679 (EGD-e), under the PCR conditions described in Materials and Methods (Section 2.3). Both the genus-specific *Listeria* primers and the *L. monocytogenes*-specific primers produced clear and specific amplification of their respective targets.

**Figure 5:**
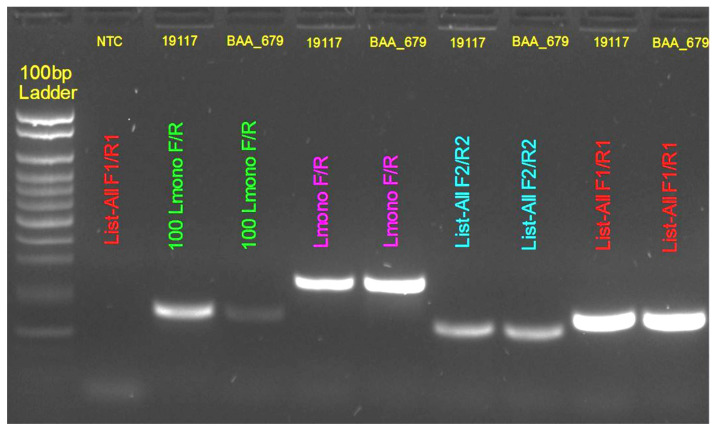
Agarose gel electrophoresis of PCR amplification products (100–300 bp). Lane 1:100 bp DNA ladder (100–1000 bp range, 100 bp increments). Lane 2: no-template control (NTC), showing absence of amplification. Lanes containing *Listeria monocytogenes* strains ATCC 19117 and ATCC BAA-679 show distinct amplification bands for genus-specific primers (red and blue labels) and species-specific primers (green and purple labels). The observed bands correspond to the expected amplicon size ranges, confirming assay specificity.

Agarose gel electrophoresis of the PCR products (Figure 5) showed distinct bands corresponding to the expected amplicon sizes within the range of approximately 100–300 bp relative to the 100 bp DNA ladder (lane 1). No amplification was observed in the no-template control (NTC), confirming the absence of contamination. Lanes containing the *L. monocytogenes* strains 19117 and BAA-679 displayed clear bands across all primer sets: genus-specific primers (blue and red labels) and species-specific primers (green and purple labels).

These observations demonstrate that the primers produced specific and reproducible amplification for both genus- and species-specific targets.

## 4. Discussion

This study provides a genome-resolved perspective on *Listeria* diversity, demonstrating that the genus exhibits pronounced genomic plasticity consistent with an open pangenome architecture [7,20]. By systematically evaluating 10 *Listeria* species across increasing ANI thresholds, we show that shared genomic content contracts sharply under stringent similarity criteria, while the accessory genome expands substantially. The reduction of the core genome from 856 genes at 60% ANI to only 16 genes at 95% ANI highlights the limited number of universally conserved loci across the analyzed *Listeria* species and suggests divergence potentially associated with adaptive processes within the genus.

The inverse relationship between core contraction and accessory expansion reflects both methodological stringency and biological reality. While conservative ortholog clustering (RIBAP) accentuates divergence by separating even moderately variable genes [19], the resulting pattern is biologically informative: it indicates that much of the genomic repertoire in *Listeria* is shaped by lineage-specific diversification, horizontal gene transfer, and ecological specialization [29]. Such dynamics are characteristic of bacteria occupying heterogeneous niches and align with prior reports describing *Listeria* as possessing an open pangenome [7,20]. Importantly, open pangenomes are not merely descriptive features; they represent evolutionary strategies that enable rapid adaptation, environmental persistence, and, in certain lineages, pathogenicity [7].

At high ANI stringency (95%), retained genes were almost exclusively associated with essential cellular functions, including translation, transcriptional regulation, and stress response [30,31]. The persistence of these loci across species suggests strong purifying selection acting on indispensable housekeeping processes [32]. Similar patterns have been documented in other taxa with open pangenomes, such as *Salmonella*, *Escherichia coli*, and *Campylobacter*, where only a minimal conserved backbone remains universally shared [33,34,35]. The extreme contraction of the *Listeria* core genome at species-level resolution therefore reinforces the concept that ecological diversification is driven primarily by the accessory gene pool rather than modifications of conserved essential machinery, as evidenced by comparative genomic analyses of *Listeria* core and accessory genome dynamics [20].

The accessory genome, which exceeded 25,000 genes at 95% ANI, appears to constitute the principal reservoir of adaptive potential within the genus. Its magnitude suggests ongoing gene acquisition and loss events, likely mediated by horizontal transfer and niche-driven selection [29]. This dynamic component shapes phenotypic diversity and may influence traits such as environmental resilience, metabolic flexibility, and virulence potential [20]. However, the sheer size and variability of the accessory gene set also complicate functional interpretation, as many genes remain uncharacterized [21].

A major contribution of this work is demonstrating that species-specific molecular markers can be reliably derived from the accessory genome, including loci annotated as hypothetical proteins. These markers exhibited high in silico sensitivity (≥90%) and specificity (98–100%), and experimental validation confirmed amplification of genus-level and *L. monocytogenes*–specific loci in two reference *L. monocytogenes* strains. These findings highlight that evolutionary exclusivity, rather than prior functional annotation, underpins the diagnostic utility of accessory genome-derived markers. Comparable patterns in other bacterial taxa support the general applicability of this genome-informed, specificity-driven approach for assay development, with potential applications in food safety, clinical diagnostics, and epidemiological surveillance [4,15,34,35].

The predominance of hypothetical proteins among species-specific genes reveals a substantial gap in our functional understanding of the *Listeria* genome. Rather than representing non-functional genetic content, these uncharacterized genes may encode niche-adaptive, resistance-associated, virulence-related, or lineage-defining functions. The success of hypothetical proteins as diagnostic markers highlights that evolutionary exclusivity, rather than functional annotation, is the primary determinant of diagnostic reliability. Similar patterns have been reported in other bacterial taxa, including *Pseudomonas* species and *Fusobacterium nucleatum*, in which species- or strain-specific hypothetical proteins were subsequently linked to cellular functions such as ecological adaptation and pathogenic potential [36,37]. Their diagnostic relevance, as demonstrated here, further indicates that functional novelty frequently coincides with genetic exclusivity. Future functional genomics investigations will be essential to determine whether these genes contribute to ecological specialization, stress tolerance, or virulence modulation [35].

In the present study, several non-pathogenic *Listeria* species were found to harbor virulence-associated genes traditionally linked to *L. monocytogenes*, including components of the *Listeria* pathogenicity island (e.g., *prfA*-associated loci) and hemolysin-related genes. Similar observations have been reported in *L. innocua*, *L. seeligeri*, and *L. welshimeri* [9,15]. The detection of these determinants outside clearly pathogenic lineages highlights a key limitation of conventional PCR assays that target classical virulence markers [4]. As noted by Naor-Hoffmann and colleagues, the presence of virulence genes in non-pathogenic strains indicates that gene possession alone does not necessarily equate to pathogenic potential [38]. This finding reinforces the need for diagnostic strategies informed by comparative genomics and evolutionary exclusivity. Accordingly, identifying truly species-exclusive targets represents a critical advancement for accurate and reliable detection.

As WGS becomes routine, species delineation using objective genomic thresholds, such as the 95% ANI, is increasingly replacing traditional phenotype-based classification systems [4]. In this context, pangenome-guided marker discovery provides a reproducible framework for designing molecular assays that accurately target both genus- and species-level taxonomic groups.

These findings highlight the accessory genome as a primary driver of genetic diversification in *Listeria* and a rich source of high-confidence diagnostic targets. By combining stringent genomic thresholds with comparative analyses and experimental validation, this study establishes a robust framework for marker discovery that goes beyond traditional virulence-based detection approaches. Such strategies are critical for improving taxonomic resolution, enhancing diagnostic accuracy, and strengthening public health surveillance in the era of routine WGS [15]. Although the curated dataset included high-quality, representative genomes across ten *Listeria* species, the inclusion of additional strains and newly described taxa may further refine marker robustness. Continuous genomic expansion of the genus may necessitate periodic reassessment of species exclusivity.

A limitation of this study was the limited availability of complete and uncontaminated genome sequences for several *Listeria* species at the time of data retrieval. Specifically, some species were represented by only a single genomic strain (*L. aquatica*, *L. newyorkensis*, and *L. weihenstephanensis*), while others had very few strains available (*L. marthii*, 2 strains; *L. grayi*, 3 strains). Furthermore, all analyzed whole-genome sequences were obtained exclusively from the NCBI database. This limited representation may affect the robustness of target identification, as intraspecies genomic diversity could influence the presence, absence, or variability of potential targets. Consequently, findings for species with only one or a few genomes should be interpreted with prudence, and future studies that include a broader range of strains could help validate and refine these targets. Additionally, ATCC strains of the other nine *Listeria* species, excluding *L. monocytogenes*, were unavailable for DNA extraction and in vitro PCR testing with their specific primers.

## 5. Conclusions

This study provides a comparative genomic analysis of ten representative *Listeria* species and identifies species-specific molecular markers derived from the accessory genome, as well as genus-specific markers derived from the core genome. The results demonstrate that lineage-specific genomic uniqueness is a critical determinant of diagnostic specificity. High in silico performance, supported by experimental validation, confirms the reliability of these loci for precise detection of *Listeria* species and *L. monocytogenes*. These findings establish a genome-informed, specificity-driven framework for molecular marker development, with potential to enhance diagnostic accuracy, epidemiological surveillance, and food safety monitoring. Future inclusion of additional *Listeria* species and newly described taxa will further refine marker robustness and broaden applicability across the genus.

## 6. Transparency, Data Availability, and Intellectual Property Statement

Whole-genome sequence data analyzed in this study are publicly available through the National Center for Biotechnology Information (NCBI) under the accession numbers listed in Table 1. All comparative genomic, pangenome, and average nucleotide identity (ANI) analyses were conducted using publicly accessible software tools and standard, reproducible workflows as described in Section 2.

Species-exclusive and genus-level diagnostic loci were identified using objective genome-based criteria, including presence across target genomes, absence from non-target genomes, and stringent sequence similarity thresholds. To ensure scientific transparency while protecting intellectual property, expanded genomic windows (±2000 bp) encompassing each diagnostic locus, along with locus lengths, functional annotation categories, and validation metrics, are provided.

Exact nucleotide sequences and primer designs derived from these loci are proprietary to QuantiPATH and are therefore not publicly disclosed. This restriction does not affect the reproducibility of the genome-based selection framework, which relies on publicly available genome assemblies, defined analytical thresholds, and traceable genomic locations. The experimental validation presented confirms the specificity and performance of representative genus- and species-level assays derived from these loci.

Requests for additional information beyond what is disclosed here will be considered in accordance with applicable intellectual property agreements. No human or animal subjects were involved in this study.

## Figures and Tables

**Table 1:** *Listeria* species and corresponding GenBank accession numbers.

*Listeria* Species	Accession Numbers
*L. aquatica*	JBDILX010000002.1
*L. grayi*	JBHUNM010000003.1
*L. grayi*	LR134483.1
*L. grayi*	UGPG01000001.1
*L. innocua*	CP071179.1
*L. innocua*	CP095723.1
*L. innocua*	CP102626.1
*L. innocua*	CP102627.1
*L. innocua*	DL083629.1
*L. ivanovii*	CP009576.1
*L. ivanovii*	CP124258.1
*L. ivanovii*	CP165991.1
*L. ivanovii*	CP165992.1
*L. ivanovii*	CP165994.1
*L. marthii*	CP089089.1
*L. marthii*	CP089090.1
*L. monocytogenes*	CP007492.1
*L. monocytogenes*	CP021325.1
*L. monocytogenes*	CP028399.1
*L. monocytogenes*	CP076127.1
*L. monocytogenes*	CP117865.1
*L. newyorkensis*	CP113980.1
*L. seeligeri*	CP034772.1
*L. seeligeri*	CP063071.1
*L. seeligeri*	CP124262.1
*L. seeligeri*	CP124268.1
*L. seeligeri*	CP148898.1
*L. weihenstephanensis*	CP011102.1
*L. welshimeri*	CP065605.1
*L. welshimeri*	CP122330.1
*L. welshimeri*	CP151430.1
*L. welshimeri*	LT906444.1
*L. welshimeri*	NC_008555.1

**Table 2:** Distribution of gene categories across ANI thresholds.

Gene Category		ANI				
		60%	70%	80%	90%	95%
Core genes		856	665	362	89	16
Soft-core genes		166	121	68	24	4
Accessory genes	Shell genes	2391	2607	3144	4339	5273
	Cloud genes	8009	9561	11,939	15,743	20,213
Total		11,422	12,954	15,513	20,195	25,506

**Table 3:** Genome-informed species-exclusive diagnostic loci identified through pangenome analysis.

Species	Internal Locus ID	Representative Genome (GenBank/RefSeq)	Expanded Genomic Window Encompassing Diagnostic Locus (bp) *	True Locus Length (bp)	Functional Category	Target Genomes Positive/Total	Non-Target Genomes Positive/Total
*Listeria (genus)*	LGEN	CP009576.1	2,835,130–2,839,348	230	Core translation-associated	100/100	0/100
*L. monocytogenes*	LMONO	CP007492.1	1,214,959–1,219,318	370	Hypothetical protein	100/100	0/100
*L. innocua*	LINN	CP071179.1	600,115–604,648	540	Hypothetical protein	22/27	5/27
*L. ivanovii*	LIVAN	CP009576.1	954,496–959,470	980	Hypothetical protein	16/16	0/16
*L. grayi*	LGRAY	LR134483.1	1,434,225–1,438,707	840	Hypothetical protein	1/1	0/1
*L. seeligeri*	LSEL	CP034772.1	423,529–427,672	144	Hypothetical protein	6/6	0/6
*L. welshimeri*	LWSH	NC_008555.1	59,202–63,405	210	Hypothetical protein	5/5	0/5

* Genomic coordinates represent an expanded ±2000 bp window surrounding each diagnostic locus and are provided to ensure genomic traceability while protecting proprietary sequence information. The actual diagnostic targets are shorter loci within these windows, as indicated by the reported true locus lengths. Exact nucleotide sequences and primer designs are not publicly disclosed due to intellectual property considerations.

## Data Availability

Data supporting the findings are available within the article.

## Abbreviations

The following abbreviations are used in this manuscript:

ANI	Average Nucleotide Identity
ATCC	American Type Culture Collection
BLAST	Basic Local Alignment Search Tool
BLEB	Buffered *Listeria* Enrichment Broth
GAIA	Genome Annotation and Information Analysis
iTOL	Interactive Tree of Life
NCBI	National Center for Biotechnology Information
NTC	no-template control
RIBAP	Roary Integer Linear Programming Bacterial Annotation Pipeline
WGS	whole-genome sequencing
